# Biofilm Specific Activity: A Measure to Quantify Microbial Biofilm

**DOI:** 10.3390/microorganisms7030073

**Published:** 2019-03-07

**Authors:** Laura Corte, Debora Casagrande Pierantoni, Carlo Tascini, Luca Roscini, Gianluigi Cardinali

**Affiliations:** 1 Department of Pharmaceutical Sciences–Microbiology, University of Perugia, 06123 Perugia, Italy; laura.corte@unipg.it (L.C.); deboracasagrandepierantoni@gmail.com (D.C.P.); gianluigi.cardinali@unipg.it (G.C.); 2 First Division of Infectious Diseases, Cotugno Hospital, 80181 Naples, Italy; c.tascini@gmail.com; 3 CEMIN-Excellence Research Center, University of Perugia, 06123 Perugia, Italy

**Keywords:** biofilm, *Candida* spp., *Staphylococcus aureus*, quantification, crystal violet, 2,3-bis-(2-methoxy-4-nitro-5-sulfophenyl)-2*H*-tetrazolium-5-carboxanilide (XTT)

## Abstract

Microbes growing onto solid surfaces form complex 3-D biofilm structures characterized by the production of extracellular polymeric compounds and an increased resistance to drugs. The quantification of biofilm relays currently on a number of different approaches and techniques, often leading to different evaluations of the ability to form biofilms of the studied microbial strains. Measures of biofilm biomass were carried out with crystal violet (CV) and a direct reading at 405 nm, whereas the activity was assessed with the XTT ((2,3-bis-(2-methoxy-4-nitro-5-sulfophenyl)-2*H*-tetrazolium-5-carboxanilide) method. The strains of four pathogenic species of the genus *Candida* (*C. albicans*, *C. glabrata*, *C. parapsilosis* and *C. tropicalis*) and of *Staphylococcus aureus* were employed to determine the effective relatedness among techniques and the specific activity of the biofilm, as a ratio between the XTT and the CV outcomes. Since the ability to form biomass and to be metabolically active are not highly related, their simultaneous use allowed for a categorization of the strains. This classification is putatively amenable of further study by comparing the biofilm type and the medical behavior of the strains.

## 1. Introduction

The microbial biofilm is a complex structure of cells not comparable to a tissue but rather to an association, also defined as a “city of microorganisms” [1]. Biofilms are formed by sessile cells growing onto biotic and abiotic surfaces [2,3] and are typically embedded in a matrix of extracellular material [4,5,6]. A unique definition of biofilm is not possible because with this term, both 3-D structures growing onto solid surfaces and floating flocs of associated microbial cells without the need of a solid substrate can be indicated [7]. Both bacteria and fungi can form biofilm not only in nature [8] but also in several anthropized environments such as food industries, where the food contact-surfaces represent a major source of contamination [9,10], or hospitals, where the tissues of patients and staff, medical equipment and other available liquid and solid surfaces can be easily colonized by biofilm [11,12]. In all these environments, this structure is considered of primary importance for the diffusion of infections and for the success of the species able to form biofilms [11,13]. The specific architecture of biofilms [14], the extracellular polymeric substance (EPS) matrix with its complex structure (polysaccharides, enzymatic components, amphiphilic compounds and other macromolecules of a different nature [15]), the nutrient limitation and slow growth, the very active or overexpressed efflux pump, the rpoS-mediated mechanisms, the genomic rearrangements such as mutations or transposition, the presence of “persister” cells and other innate or induced mechanisms infer an increased resistance to stressing agents including drugs [16,17,18,19,20,21]. Biofilm detection and analysis can be carried out with a variety of methods, each based on a specific reaction of the cells or of the extracellular matrix.

Crystal violet (hereinafter referred as CV) is one of the most popular and is based on the ability of this dye to color the polysaccharidic matrix [22,23]. The XTT ((2,3-Bis-(2-Methoxy-4-Nitro-5-Sulfophenyl)-2*H*-Tetrazolium-5-Carboxanilide) procedure highlights the metabolic activity via the reduction of tetrazolium salts to formazan [24,25]. Also, the absorbance of light at 405 nm [26] and other innovative procedures [8,27] are present in the armory of the microbiologist. All these procedures carry out the specific measure of biofilm presence or the activity of the cells attached to a surface after repeated washings. More difficult is the direct quantification of the number of cells composing a biofilm, due to the complex structures created during its growth. One solution to this problem was proposed by Heydorn and colleagues who developed a software to try to calculate several parameters from three dimensional clusters of biofilm images [28]. Another way is the one proposed by Bakke et al. or Casagrande et al., which tried to use an optical approach to quantify the biofilm [29,30]. In general, most of the methods can be divided into two categories according to the ability to measure the biofilm biomass as CV or its metabolic activity as XTT. Although these two types of measures are not necessarily correlated, the literature is using the various methods as alternatives amenable to give comparable results [31].

Ramage and colleagues noted that there is little correlation between the CV and XTT measures and decided to use only the former during a survey of *Candida* biofilms isolated in Scotland [32]. Similarly, Xu and coworkers had similar results working with a large collection of *Staphylococcus aureus* strains [33]. Even if, in literature, some examples of the comparison of different biofilm detection assays are reported [34,35,36], to our knowledge, there is not, at this time, a systematic analysis of the relation between these two analytical techniques in both bacteria and fungi. This aspect is of potential interest because both bacteria and yeast are analyzed for the biofilm production with little or no standardization. The study described in this paper started from the concept that CV and XTT measures of biofilm formation are not only poorly correlated [32,37] but can be considered indeed two independent descriptors of biofilm biomass and activity in both bacteria and fungi, as also demonstrated by several experimental evidences obtained during the setup of this work. Furthermore, the independence of these descriptors can be used to develop a series of standardized metrics for a better and comparable characterization of the biofilm-forming strains. For this purpose, we used a series of yeast strains belonging to *Candida albicans*, *C. parapsilosis*, *C. tropicalis* and *C. glabrata*. Similarly, thirty-one isolates of *S. aureus* were identified and characterized with the same technique. This approach is amenable of being used with both bacteria and fungi and gives also the opportunity to evaluate the synergy of fungal–bacterial interactions found in nature and in clinics [38].

## 2. Materials and Methods

### 2.1. Strains and Growth Conditions

Thirty strains belonging to the four major pathogenic species of the *Candida* genus (*Candida albicans* CMC 1768, CMC 1778, CMC 1818, CMC 1829, CMC 1845, CMC 1913, CMC 1968, CMC 1986, CMC 2020, CMC 2042, CMC 2049 and CMC 2053; *Candida parapsilosis* CMC 1838, CMC 1841, CMC 1859, CMC 1949, CMC 1972, CMC 2012, CMC 2039 and CMC 2050; *Candida tropicalis* CMC 1798, CMC 1827, CMC 1855, CMC 1904, CMC 1961, CMC 1978, CMC 2017 and CMC 2052; and *Candida glabrata* CMC 1830 and CMC 2032) and thirty-one strains belonging to *Staphylococcus aureus* species (PI 850, PI 3024, PI 3025, PI 4827, PI 5010, PI 5013, PI 5095, PI 5194, PI 5319, PI 5379, PI 5398, PI 5540, PI 5616, PI 5639, PI 5656, PI 5696, PI 5920, PI 6163, PI 6177, PI 6427, PI 6499, PI 6500, PI 6624, PI 6667, PI 6668, PI 6749, PI 6869, PI 6870, PI 7090, PI 7091 and PI 7092), all isolated in a clinical (medical) environment, were employed in this study. Yeast strains were isolated and identified as described by Corte et al. [11], bacterial strains were all isolated from diabetic foot in Pisa hospital and identified by rDNA analysis and MALDI-TOF mass spectrometry.

The isolates were kept frozen at −80 °C in 17% glycerol. Short term storage was carried out at 4 °C on YEPDA (Yeast Extract 1%, Peptone 1%, dextrose 1% (YEPD) added with 1.7% agarose) for yeast strains and BHI (Brain Heart Infusion) for bacterial strains. The yeast cells were grown in YEPD (all products from Biolife—http://www.biolifeitaliana.it/) at 37 °C with 150 rpm of shaking; bacterial strains were grown in BHI (Biolife—http://www.biolifeitaliana.it/) in the same conditions used for yeasts.

### 2.2. Biofilm Assay

Biofilm presence and activity were assessed with colorimetric methods based on crystal violet and XTT reactions [25,39], with slight modifications. Briefly, each strain was grown over night in bottles containing the appropriate medium (YEPD for yeasts and BHI for bacteria) at 30 °C in an orbital shaker at 150–180 rpm and then harvested and centrifuged at 3000× *g* for 5 min at 4 °C. The supernatant was removed, and the pellet was washed twice with Phosphate Buffered Saline (PBS). The washed cells were then resuspended in an RPMI-1640 medium (Sigma Aldrich, Saint Louis, MO, USA, used for yeasts) or TSB (Tryptic Soy Broth, Biolife, Milan, Italy, used for bacteria) in order to obtain a final density of 1.0 × 10^6^ cells/mL, by adjusting the density after spectrophotometrical readings at OD_600_ and the calculation with the regression equation of the species-specific curves [30]. One hundred µL of this standardized cell suspensions were seeded in each selected well of a 96-well microtiter plate; one unseeded well acted as the negative background control for the subsequent steps. Three different replicates for each strain were set into each plate. Two different plates for yeasts and two for bacteria were prepared. These microtiter plates were then closed, sealed and incubated for 2 h at 37 °C. After biofilm surface priming, the medium in each well was removed carefully with a multichannel pipette, taking care to not disrupt the biofilm; each well was subsequently washed three times with PBS. At this stage, it was not possible to spectrophotometrically evaluate the biofilm formation because its optical density was under the detection limit of the plate reader [30]. After these washing steps, 100 µL of the appropriate medium was added to the wells. Each plate was then closed, sealed again and incubated for 24 h at 37 °C to permit the biofilm development. The plates were then recovered, and the same washing procedure described above was applied. One plate for yeasts and one for bacteria was stained with 1% solution of crystal violet (100 µL each well) for 15 min; they were then washed three times with water and dried at room temperature, and the absorbance of adherent biofilm cells was measured with a TECAN Infinite F200 plate reader (Tecan Trading AG, Mannedorf, Switzerland) at 570 nm. The second plate prepared for yeast and bacteria was stained with a XTT/menadione solution prepared as described by Pierce and colleagues [25] (100 µL each well); the biofilm activity was then measured with a TECAN Infinite F200 plate reader at 492 nm. Each strain was tested for biofilm production in triplicates, and the assay was repeated three times.

### 2.3. Data Analysis

Densitometric data were recovered from the TECAN interface and transferred to MS Excel. Given the popularity of this software, we prepared a simple template to automatize the analysis of each plate. The template contains three 8 × 12 layouts, reporting the disposition of strains and conditions in 96 well plates, the readings with CV and the ones with XTT, respectively. A summary table in the same sheet reports the statistical analyses and gives an automatic definition of the ability of forming biofilm based on the Student’s *t*-test. The experiments described in this paper had three technical and three biological replicas. In each plate, the three technical replicas were placed in three sectors of four columns, each including 30 strains and two control wells. The CV and XTT values were normalized according to Equation (1) and Equation (2) to determine the normalized values of CV and XTT, CV_N_ and XTT_N_ respectively,

CV_N_ = (CV − Cont_CV_)/Cont_CV_ (1)

XTT_N_ = (XTT − Cont_XTT_)/Cont_XTT_ (2)

where CV and XTT are the averages of the three readings and Cont_CV_ and Cont_XTT_ are the averages of the readings of the control wells without cells. This procedure normalizes the data, allowing for a good comparison among different experiments and taking into consideration the various effects (e.g., little variations in the dye solution, different plastics of the plates, etc.) that can influence the reading values. The data are then used to calculate the XTT to CV rate (XCR), the Biofilm Specific Activity (BSA) and the Biofilm Metabolic Volume (BMV), according to Equations (3)–(5).

XCR = XTT_N_/CV_N_ (3)

BSA = (XTT_N_/CV_N_) × [(CV_N_ + XTT_N_)/2] (4)

BMV = XTT_N_ × CV_N_ (5)

The XCR parameter is a measure of the metabolic activity referring to the biofilm biomass and suffers of producing high values when the CV_N_ is relatively low. It was, therefore, changed in the BSA by multiplying the XCR by the average of the XTT_N_ and CV_N_, obtaining a descriptor of the XCR weighted on the average of the CV and XTT values. Finally, the BMV combines both the activity and biomass of the biofilm matrix in a single metric.

### 2.4. ROC Curves Calculation

Receiver Operating Characteristics (ROC) curves for each descriptor were calculated with an MS Excel template. For each chosen threshold value of the descriptors, four metrics are calculated according to Equations (6)–(9).

True Positive Rate = Σ True Positive/(Σ Condition Positive)(6)

False Positive Rate = Σ False Positive/(Σ Condition Negative)(7)

Accuracy = (Σ True Positive + Σ True Negative)/Σ Total population(8)

Specificity = Σ True Negative/(Σ Condition Negative)(9)

The Sensitivity parameter coincides with the True Positive Rate because the greater the sensitivity of the test, the better will be the ability of identifying True Positive observations.

## 3. Results

### 3.1. Crystal Violet and XTT as Independent Descriptors of the Biofilm Forming Ability

**Figure 1:**
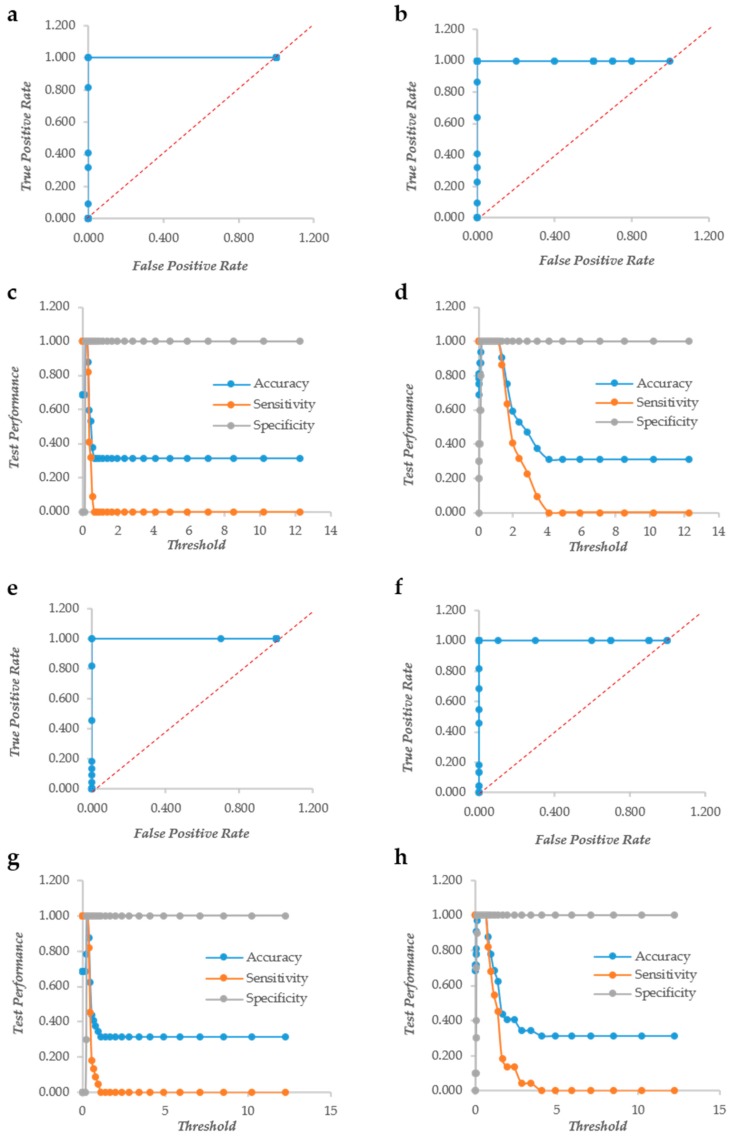
The Response Operator Characteristic (ROC) curves showing the ability to classify raw and normalized CV and XTT TECAN reads of *Candida* biofilm. The ROC curve and its parameters were obtained from raw read values recorded for CV staining (Panel (**a**) and (**c**) respectively), from normalized read values calculated for CV staining (Panel (**b**) and (**d**) respectively), from raw read values recorded for XTT staining (Panel (**e**) and (**g**) respectively) and from normalized read values calculated for XTT staining (Panel (**f**) and (**h**) respectively).

The biofilm formation of 31 strains of *Staphylococcus aureus* and 30 pathogenic yeasts of the genus *Candida* was detected using both crystal violet (CV) and XTT. The color formation was observed visually to define the biofilm forming ability of the bacterial and yeast strains. In particular, the yeast biofilm data confirmed the results on biofilm formation previously published [11]. The same plates were read in triplicate with a TECAN plate reader at 570 and 492 nm for CV and XTT, respectively. One of the major problems when using accurate reading is to establish thresholds to define when a given value of CV or XTT indicates biofilm formation. The change of the threshold level influences also the yield of false positives and negatives, as displayed in ROC curves. For these reasons, we used the row and normalized values of the CV and XTT readings to produce ROC curves and studied the trend of accuracy, sensitivity and specificity with the change of the threshold value. In *Candida* spp., crystal violet (CV) behaves as a “perfect” descriptor (i.e., a classifier able to produce a perfect discrimination between True Positives and True Negatives without False Positives or Negatives) with and without normalization (Figure 1a,b). However, the accuracy and sensitivity non-normalized metric drops at very low values of the threshold, whereas they remain stable up to threshold 1.3 with the normalization. (Figure 1c,d). XTT, as well, is a “perfect” descriptor according to the ROC curves of both normalized and not normalized data (Figure 1e,f). The sensitivity and specificity show a larger threshold range in normalized data, although less evident than in CV (Figure 1g,h).

**Figure 2:**
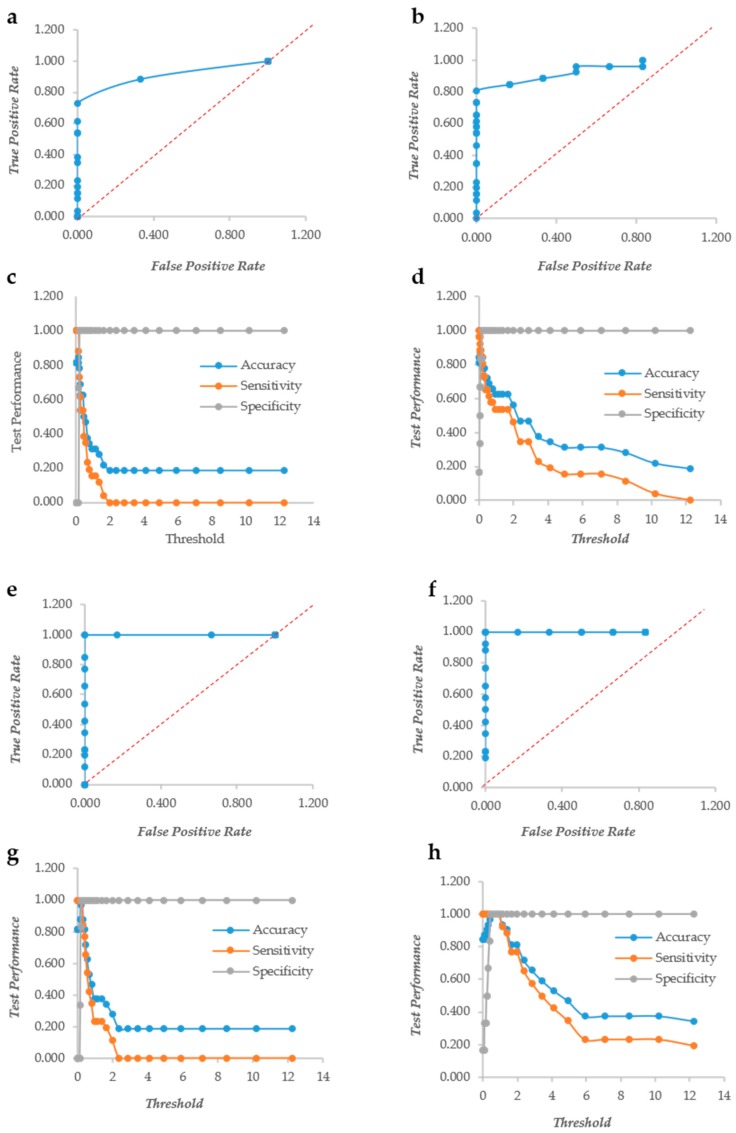
The Response Operator Characteristic (ROC) curves showing the ability to classify raw and normalized CV and XTT TECAN reads of *Staphylococcus aureus* biofilm. The ROC curve and its parameters were obtained from raw read values recorded for CV staining (Panel (**a**) and (**c**) respectively), from normalized read values calculated for CV staining (Panel (**b**) and (**d**) respectively), from raw read values recorded for XTT staining (Panel (**e**) and (**g**) respectively) and from normalized read values calculated for XTT staining (Panel (**f**) and (**h**) respectively).

In *S. aureus*, CV does not display the ROC curves of a “perfect” descriptor although the curves are quite distant from the diagonal, indicating a very good descriptor (Figure 2a,b). The sensitivity and accuracy decreased steeply without a plateau as observed in yeasts. The normalization, however, made the decrease of these two indicators less steep; in fact with a threshold = 4, the accuracy of the non-normalized indicator was 0.2, whereas that of the normalized counterpart was 0.344. Similarly, the sensitivity at threshold = 4 were 0.0 and 0.192 for the non-normalized and the normalized data, respectively (Figure 2c,d). The XTT behavior is more similar to that observed in yeasts; in fact, the ROC curves are typical of a “perfect” descriptor (Figure 2e,f), and the accuracy, sensitivity and specificity remain at their maximum up to a 1.3 threshold with normalized data, whereas the non-normalized decreased steeply without any plateau (Figure 2g,h). These findings indicate that indeed the normalization plays a role with both CV and XTT either in *Candida* or in *Staphylococcus*. The two descriptors resulted rather independent, as demonstrated by the low Pearson correlation r values: 0.68 and 0.72 in *Candida* and *Staphylococcus*, respectively. These figures are quite comparable with those obtained in different studies [32] and show that the two assays give different types of information on the microbial biofilm. CV is known to be a good indicator of the amount of biomass, while XTT is bound to the activity of the cells forming the biofilm.

### 3.2. CV and XTT Work Together with Derived Indexes

**Figure 3:**
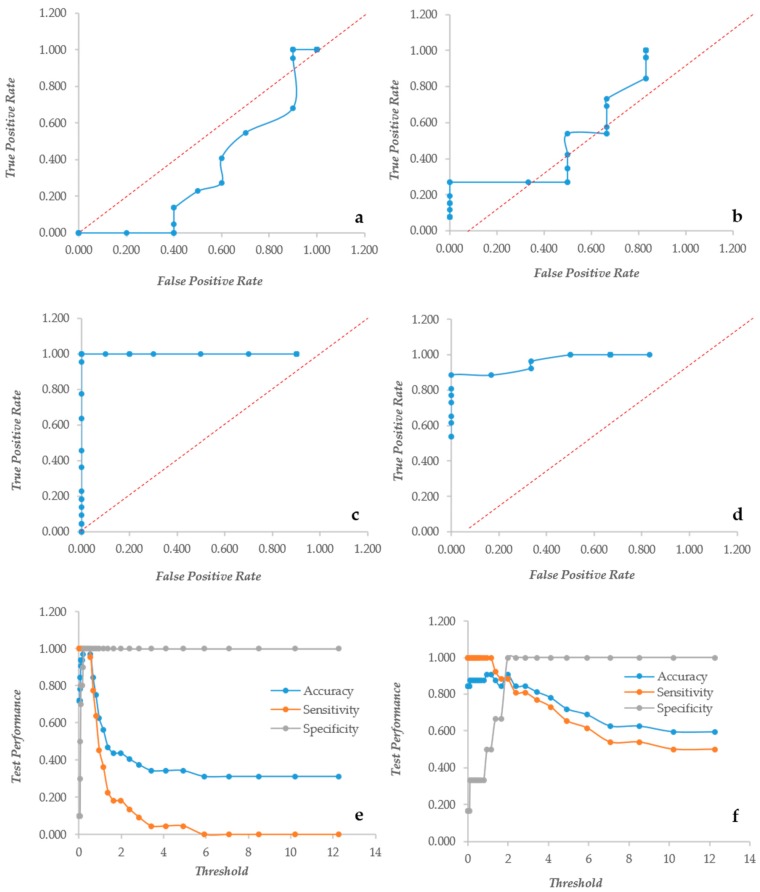
The Response Operator Characteristic (ROC) curves showing the ability to classify normalized XTT to CV rates (XCR, panel (**a**) and (**b**)) and Biofilm Specific Activity (BSA, panel (**c**–**f**). Panels (**a**) and (**b**): the ROC curves for the XCR values of *Candida* and *Staphylococcus* respectively; panels (**c**) and (**e**): the ROC curve and its parameters for the BSA values of *Candida* biofilm; and panels (**d**) and (**f**): the ROC curve and its descriptors for the BSA values of *Staphylococcus* biofilm.

Since the two assays bring different types of information, their ratio XCR (XTT over CV Ratio) is expected to indicate how much metabolic activity is present per unit of biomass, as detected by the CV assay. Unfortunately this index had a very poor performance in both yeast and bacteria, as shown by the ROC curves mostly under the diagonal line (Figure 3a,b). Furthermore, the ratio suffered extremely high values when the CV was relatively low, producing very high figures. In order to fix these problems, the XCR was multiplied by the average of the CV and XTT values, obtaining an index called BSA (Biofilm Specific Activity) with better performances and without the problems related to low CV values. In *Candida*, BSA showed the typical ROC curve of a “perfect” descriptor (Figure 3c). Even in *Staphylococcus*, the ROC was an almost “perfect” descriptor (Figure 3d) with better than that displayed by the CV alone. BSA in *Candida* displayed a very high specificity at any threshold, whereas the sensitivity and accuracy remained high up to the threshold 0.662 (Figure 3). In *Staphylococcus* instead, the specificity increased from 0.2 to 1 with thresholds respectively of 0 and 2. The accuracy and sensitivity decreased extremely slowly that a threshold = 4 displayed values of 0.78 and 0.73, respectively (Figure 3f).

**Figure 4:**
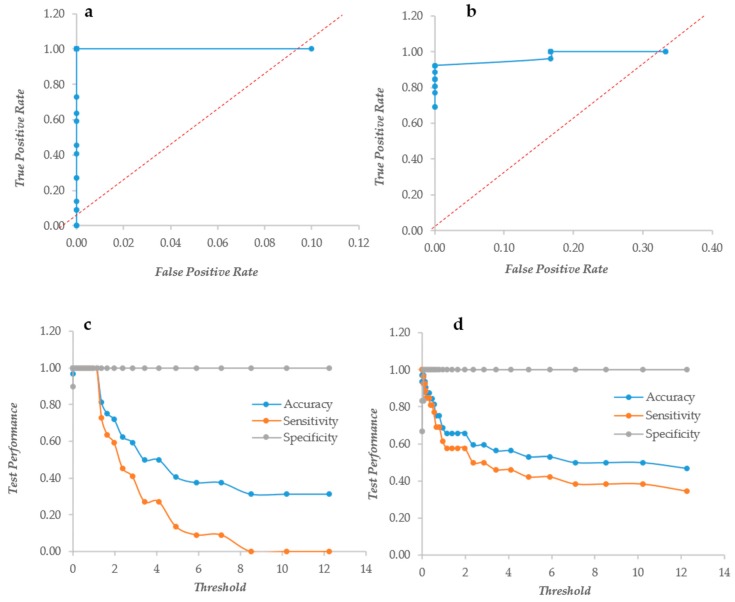
The Response Operator Characteristic (ROC) curves showing the ability to classify XTT and CV value products. Panels (**a** and **b**): the ROC curves for the CV and XTT product values of *Candida* and *Staphylococcus* respectively; panels (**c** and **d**): the ROC curve parameters for *Candida* and *Staphylococcus* biofilm respectively.

Another index to put together the information of XTT and CV was the Biofilm Metabolic Volume, resulting from the product of the XTT and CV data. Once again, this index had the ROC of a “perfect” descriptor in *Candida*, whereas it performed less in *Staphylococcus* (Figure 4a,b). In *Candida*, the three performance parameters remained at their maximum up to a threshold of 1.37 (Figure 4c), whereas in *Staphylococcus*, there was a less steep descent of the accuracy and sensitivity that showed figures of 0.56 and 0.46 at threshold 4 (Figure 4d).

### 3.3. Crystal Violet and XTT Used Jointly to Classify Different Types of Biofilm

**Figure 5:**
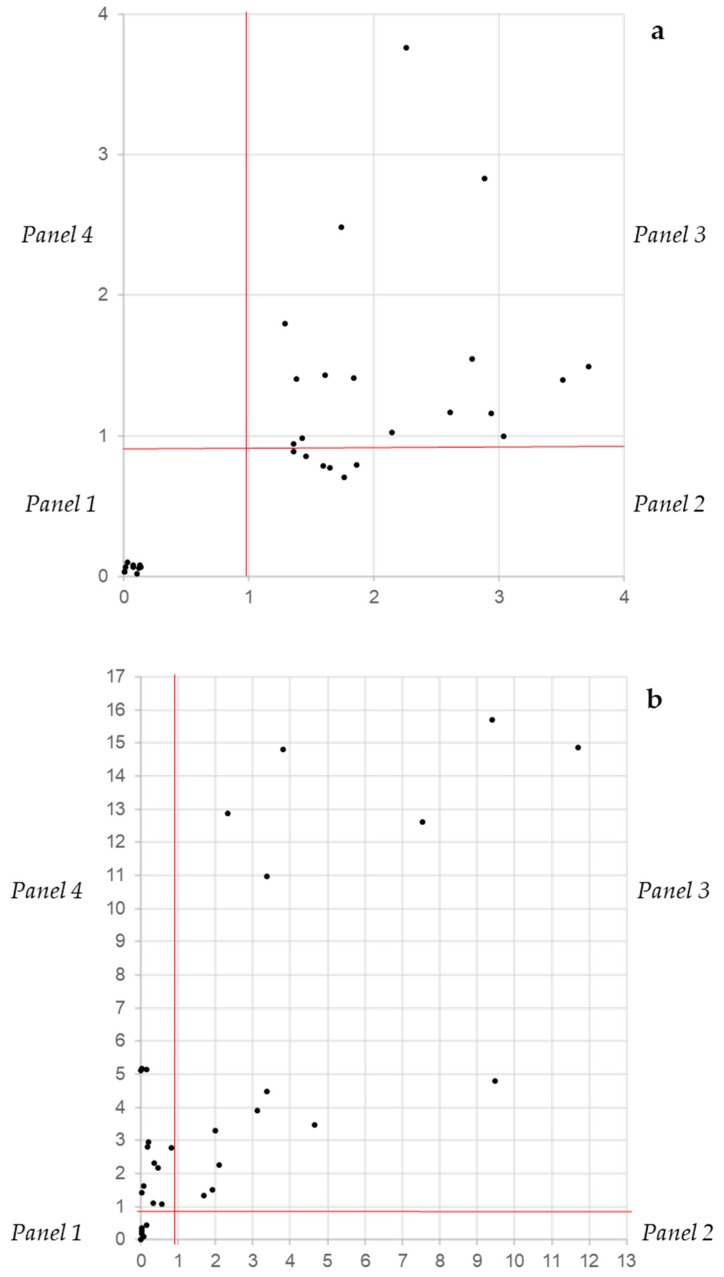
The distribution on a bidimensional space of *Candida* (**a**) and *Staphylococcus* (**b**) strains according to their XTT_N_ and CV_N_ values. Panel 1: No biofilm production, panel 2: biomass production and low biofilm activity, panel 3: biomass production and high biofilm activity and panel 4: low biomass production and low biofilm activity.

The independence between the CV_N_ and XTT_N_ assays gives the opportunity to classify biofilm producers into four categories according to their distribution in a Cartesian bidimensional space. Considering the performances of the two descriptors and the range of the threshold at which all three performance parameters are at their best, the threshold was set at 1, meaning that values below 1 indicate a poor if any metabolic activity (XTT_N_ on the y axis) and a low biomass (CV_N_ on the x axis). Since the CV_N_ and XTT_N_ values derive from a normalization, this threshold is not expected to suffer for different settings, as the overall background readings. The separation at threshold 1 of both axes produced four panels designated as no biofilm (panel 1: lower left), inactive producers (panel 2: lower right), active producers (panel 3: upper right) and active low producers (panel 4: upper left). In *Candida*, the strains that were known as no biofilm producers clustered all in panel 1 very close to the origin (Figure 5a). The rest of the strains were scattered throughout panels 2 and 3, and none was found in panel 4. Vice versa, in *Staphylococcus* sp., strains were present in panels 1, 3 and 4 (Figure 5b). These data suggest that, in general, the *Staphylococcus* biofilm is more active.

## 4. Discussion

An analysis of the microbial biofilm can be carried out with a number of different assays, including crystal violet and XTT methods [25,39]. The detection of the biofilm in vitro can be performed visually by experienced personnel or automatically with multi-well plate readers. The former system is affected by the sensitivity and experience of the analyst, whereas the latter produces continuous data of absorbance that must be interpreted. In the case of a “yes-or-not “output, a single threshold must be chosen in order to discriminate the positive over the threshold from the negatives below. In other cases, it has been chosen to divide the range of values in quartiles [32], leading to a more articulated description of the strains according to their ability to produce biomass, if CV is used or to be metabolically active, according to the XTT assay. The lack of normalization could cause scarce reproducibility among laboratories and sometimes even among different experiments within the same laboratory. Changes of the absolute values of the readings could be due to different absorbances of the plates, to different levels of nonspecific binding of the dye (particularly CV) to both the plastic of the multi-well plate and microbe cells wall and to different energies of the lamp or absorbance of the filter. All these factors can change significantly the background and, therefore, the actual reading of the biofilm. One possibility is simply to subtract the background from the readings, the other is the normalization presented in this paper.

There is an obvious linear relationship between the results of the two corrections, and in both cases, readings at the background level will be transformed in 0. However, the normalization has the advantage of expanding the range of values at the rate of the inverse of the background. For example, with a background level of 0.125, the range of the normalized values will be expanded by 1/0.125 = 8 times. This enlarges the differences between the negative and the positive results, easing the search of an optimal threshold and, in general, the analysis of the data. The fact that both CV and XTT behave as “perfect” descriptors justifies their large use as witnessed by some 25,000 and 3500 citations in the literature for these two assays respectively. However, the problem of using either of the two assays is that each of them describes the biofilm differently. This makes biofilm data not fully comparable among different papers. In fact, some will describe the biofilm as for its ability to form biomass and others for its metabolic activity.

The solution proposed in this paper is to produce two derivate indexes of which one, the BSA, indicates the specific activity of the biofilm and the other, the BMV, is a sort or complex score of the biofilm ability to grow and be active. The BSA is potentially useful when the devitalization of the biofilm is under study, i.e., in those cases in which the biomass is not supposed to vary, but the activity should decline due to the effect of the devitalizing agent. On the other hand, the BMV can be a rapid index to assess the real effectivity of the biofilm and, therefore, its threat in the environment and in the patients. The classification into four classes presented in this paper is based on the combination of the activity and ability to produce biomass with a single threshold value at 1.00. It can be made more complex and informative by setting more than one threshold level and, for instance, dividing the CV and XTT range in four quartiles, applying to each assay the procedure proposed for the CV alone [32]. It is obvious that under the pressure of a rapid diagnosis, the question is simply whether or not the cells produce a biofilm. This means that in hospital settings, only one of the two assays could be considered enough to decide on the therapy. However, the ability of the biofilm to resist drugs [6,40,41] and to be the most important factor for the survival of the species in harsh environments [11,42,43] requires a deeper knowledge of its features for a full understand of its dynamics and, therefore, for the development of fully rationale responses aiming at its limitation or management.

## 5. Conclusions

The analysis of microbial biofilm formation can be carried on with a variety of methods, most of which based on automatic readers. In this paper we have demonstrated that the normalization, with the introduction of synthetic indexes, can lead to significant benefits in the evaluation of biofilm biomass and activity. Furthermore, we showed that it is possible to take advantage of the statistical independence of the biomass evaluation with XTT or CV, by producing a distribution of the strains with both synthetic indexes, with an improvement of the resolution obtainable among strains. Future studies could be helpful to add other descriptors for a better description of biofilm-forming strains.
